# Internationally Educated Nursing Staff Caring for Older Adults: A Scoping Study

**DOI:** 10.1177/10436596241239300

**Published:** 2024-03-28

**Authors:** Sherif A. Olanrewaju, Susan J. Loeb

**Affiliations:** 1 The Pennsylvania State University, University Park, USA

**Keywords:** Internationally Educated Nursing staff (IENs), older adults, long-term care setting, Scoping studies

## Abstract

**Introduction:**

Internationally Educated Nursing staff (IENs) are born and obtained their initial educational preparation in their home country before relocating to work in high-income countries (e.g., United States). Older adults are recipients of IENs’ care. The study purpose was to synthesize relevant findings on IENs’ experiences caring for older adults in various settings.

**Method:**

Arksey and O’Malley’s framework approach to Scoping studies was employed. The PubMed, CINAHL, PsycINFO, Web of Science, and Google Scholar databases were searched. A labor and employment relations researcher and a health science librarian were consulted.

**Results:**

Three main themes (transitional challenges; IENs’ experiences working with older adults; factors affecting IEN service delivery) and seven subthemes emerged.

**Discussion:**

Cultural beliefs and communication barriers posed particular challenges to IENs as they worked with Western peers, older adults, and families. Facilitating factors revealed can inform administrative leaders of practice initiatives. Research gaps and limitations identified can guide future study approaches.

## Introduction

The global nursing shortage has prompted a surge in the migration of nurses—especially from countries with limited resources to high-income nations like the United States, Canada, the United Kingdom, and Australia. The term “Internationally Educated Nursing staff” (IENs) refers to those who completed their initial nursing education in their native countries before moving to work as nursing staff in high-income countries (Schumacher, 2011; Viken et al., 2018; Walani, 2015). For the purpose of this review, nursing staff refers to migrants practicing as registered nurses (RNs), licensed practical nurses (LPNs)/licensed vocational nurses (LVNs), certified nursing assistants (CNAs), and nursing assistants (NAs) in high-income countries. According to the Organization for Economic Co-operation and Development (2021), the high demand for nurses worldwide has contributed to substantial increases in the rate at which nurses are migrating, particularly from resource-constrained countries to high-income ones. Western countries have responded to their nursing deficits by recruiting IENs (Li et al., 2014). Many prosperous countries now have immigration policies designed to motivate nurses to immigrate there to work (Masselink & Jones, 2014). These changes, instituted since the 1980s, have led to a significant increase in the inflow of IENs into the nursing workforce in high-income countries, temporarily alleviating the shortage of nursing staff (England, 2015; Masselink & Jones, 2014). IENs are found throughout various health systems, yet they are significantly overrepresented in long-term care (LTC) settings (Wagner, Brush, Engberg, et al. 2015). Furthermore, compared to U.S.-educated nurses, IENs tend to have higher retention rates in the workforce (Rapp & Sicsic, 2020). This trend suggests that the reliance of the United States on the contributions of IENs is likely to continue (Thompson et al., 2022).

Li et al. (2014) classified issues driving IEN migration as pull and push dynamics. The pull factors from recipient countries include opportunities for attractive remuneration, professional development, personal growth, recognition of professional expertise, and an improved standard of living, as well as labor-friendly employment policies, professional autonomy, a steady socio-political environment, and excellent retirement benefits. The push factors from source countries are basically the opposite of the pull factors (Li et al., 2014).

IENs often face difficulties when integrating into the nursing workforce. Shaffer and Dutka (2013) identified language proficiency, varying standards of nursing education across different countries, and regulatory hurdles as significant challenges for IENs. The costly and complex process of obtaining a nursing license, cultural barriers, and discrimination can present substantial obstacles for IENs (Wolcott et al., 2013). Once in the United States, many IENs face social isolation, cultural differences, difficulties adapting to a new lifestyle, the need to understand new health care technologies (Jose, 2011), difficulty developing new relationships, discrimination, and low self-esteem. In addition, they may recognize that they received insufficient education and training in their home countries (Ghazal et al., 2020; K. Iheduru-Anderson, 2020; K. C. Iheduru-Anderson et al., 2021; Rosenkoetter et al., 2017; Walani, 2015).

As per data from 2016, the United States was home to 49.2 million older adults, making up 15.2% of the total population (Roberts et al., 2018). With older adults requiring three to five times the health care of younger people (Roberts et al., 2018), the growing aging population will significantly impact health care delivery, especially with a shift from acute to chronic illness and a potential shortage of health care workers (Wiener & Tilly, 2002). By 2030, it’s expected that 70% of all nurses will care for an older adult (Roberts et al., 2018), the demographic most likely to be hospitalized. Regrettably, the U.S. health care system is unprepared to handle the increased number of older adults, and this issue was aggravated by the onslaught of the COVID-19 pandemic (Fulmer et al., 2021). The investigators recommended that the U.S. health care system implements several stringent measures to promote adequate health care for the growing older population. One of these measures is expanding the nursing workforce. Fulmer et al. (2021) suggested that the nursing workforce should be prepared to achieve patient-centered care. Donelan et al. (2019) contended that the U.S. health care system should recognize the needs of older adults. As well, the nursing workforce should be heterogeneous and more nurses should be recruited to promote heterogeneity (Fulmer et al., 2021). The accelerating aging of the population is escalating the need for RNs in LTC settings. The migration of IENs plays a crucial role in mitigating the nursing shortages that accompany this demographic trend (Xiao et al., 2014). IENs have a fundamental role in transforming health care in LTC settings by providing a value-driven and high-quality environment for older adults living in such settings (Tomlinson et al., 2020).

Previous studies have examined the working experiences of IENs in high-income countries (Viken et al., 2018; Zhong et al., 2017); however, this study focuses specifically on experiences of IENs caring for older adults in high-income countries. The purpose of this article is to conduct a Scoping study of the literature to determine what evidence exists on IENs’ experiences caring for older adults and to synthesize study findings.

## Methods

Scoping study methodology was used to ensure comprehensive coverage of the available literature on IENs’ experiences caring for older adults. This Scoping study was governed by the “Preferred Reporting Items for Systematic Reviews and Meta-Analyses Protocols Extension for Scoping Review” (PRISMA-ScR). Scoping studies provide a detailed description of the available findings to ascertain the breadth of research in this area and identify research gaps in the existing works of literature (Arksey & O’Malley, 2005). In this review, we followed Levac et al.’s (2010) recommendations, based on the Arksey and O’Malley’s (2005) framework for conducting a Scoping study. The process is divided into at least five stages with an option for a consultation exercise as the sixth stage. The stages are: (a) identifying the research question; (b) relevant studies; (c) selecting studies; (d) charting the data; (e) collating, summarizing, and reporting the results; and (f) consultation (Arksey & O’Malley, 2005; Levac et al., 2010). The six stages are applied below.

### Identifying the Research Question and Eligibility Criteria

The objectives are to guide additional research by identifying gaps in the literature for future studies. This article will address the following review questions:

1. What are the experiences of IENs caring for older adults?
2. What factors facilitate or impede IENs’ provision of high-quality health care services to older adults?

#### Inclusion Criteria

The following inclusion criteria were applied throughout the process. The empirical studies that were selected for review examined IENs directly caring for older adults in LTC settings, including but not limited to nursing homes, hospice care, and assisted living. Articles included reports of original research that were available in English, and were published from 2000 to May 2023. The period covered by the publications was limited to the last two decades when nursing was designated as a “shortage occupation” and the United States began issuing permanent resident permits to IENs in the 2000s (Masselink & Jones, 2014). Inclusion criteria were verified through the title and abstract.

#### Exclusion Criteria

Exclusion criteria include articles published in a language other than English, studies conducted in acute care settings or that do not primarily provide care for older adults, conference abstracts, comments, and gray literature such as dissertations and information posted on organizational websites. Also, articles reporting on syntheses of existing literature, such as systematic, scoping, or integrative reviews studies, were also excluded.

### Identifying Relevant Studies

Five online databases, PubMed, CINAHL, Web of Science, PsycINFO, and Google Scholar, were searched for this review. The initial search of PubMed was conducted using the following keywords: (((“Nurses, International”[Mesh]) OR (internationally educated nurse*[Title/Abstract] OR IEN[Title/Abstract] OR migrant nurse*[Title/Abstract] OR immigrant nurse*[Title/Abstract] OR foreign nurse*[Title/Abstract])) AND (((“Nursing”[Mesh]) OR “Geriatric Nursing”[Mesh]) OR (caring[Title/Abstract] OR nursing[Title/Abstract] OR providing care[Title/Abstract] OR geriatric care[Title/Abstract])) NOT ((“Breast Feeding”[Mesh]) OR (breastfeeding OR “breast feeding”)) AND ((“Aged”[Mesh]) OR (older adult*[Title/Abstract] OR aged[Title/Abstract] OR elderly[Title/Abstract] OR senior[Title/Abstract] OR older people[Title/Abstract] OR geriatric[Title/Abstract]))). The subsequent phase involved initiating the search across the remaining four databases, using tailored search strings that were adjusted to align with unique parameters of each database. A subject expert in Labor and Employment Relations and a health sciences reference librarian were consulted. The latter validated the keywords and search strategies.

### Study Selection

In October 2021, a preliminary search in PubMed yielded 32 potential citations. A similar search was expanded to CINAHL, Web of Science, PsycINFO, and Google Scholar, which yielded 62, 76, 49, and 202 articles, respectively. In addition, the reference lists of the retained articles were examined, revealing six additional relevant studies. Gray literature was not sought for inclusion in this Scoping study; rather, it served to furnish contemporary context and potential insights regarding IENs and older adults, which facilitated discussion in relation to scholarly literature. The first author made the initial study selection. Two stages were involved in the study selection process. First, the authors conducted independent reviews of the titles and abstracts and shared findings to determine their initial selection for inclusion. In the second stage, full-text reviews were completed, whereby the authors determined if the articles met the inclusion criteria. Articles that did not meet all requirements were removed.

### Charting the Data (PRISMA Figure 1)

**Figure 1.**
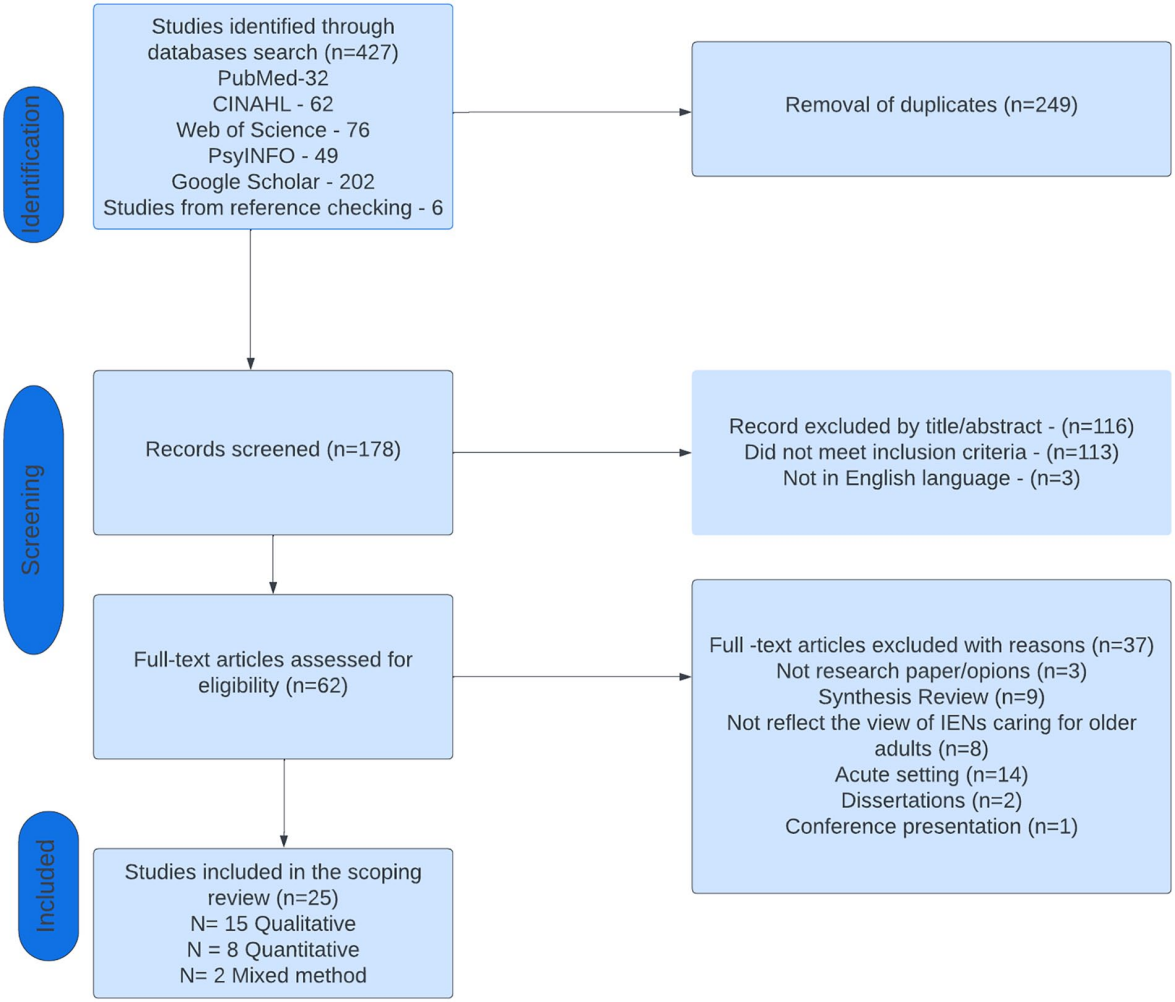
PRISMA Flowchart. *Note.* IENs = Internationally Educated Nursing staff; PRISMA = Preferred Reporting Items for Systematic Reviews and Meta-Analyses.

Most of the database searches were conducted in October 2021 and May 2023. Overall, 427 articles were retrieved from five databases and through reference list checking. Using the Zotero reference manager, 249 duplicates were removed. After checking the titles and abstracts of the remaining 178 articles to eliminate those that appeared to be irrelevant (i.e., did not meet inclusion criteria and/or were not in the English language), the total number of articles was reduced to 62. After reading the full text of the 62 remaining articles, 37 articles were removed, leaving 25 articles to be included in the Scoping study. The first author reviewed every article retrieved from the databases, while all both collectively read and deliberated on the articles deemed suitable for final inclusion. The PRISMA flowchart diagram depicting the articles included in the search process is shown in Figure 1.

### Collating, Summarizing, and Reporting the Results

Table 1 presents the detailed list of selected articles, their characteristics, and contributions to the findings. The literature was abstracted using Garrard’s Matrix method (Garrard, 2020). This data matrix facilitated the organization of data from the 25 selected studies. This matrix (Table 1) provides a description of the 25 articles which covers the following significant areas: authors/year of publication, country, study aims, methods, setting, participants/sample size, relevant findings, and strength and weakness of the articles. The foundation for initial data charting was established through this process. An intensive review of the collated data pinpointed recurrent themes, patterns, and insights that were germane to the study’s guiding questions. The themes that were relevant to the research questions were extracted from the eligible articles. Each primary theme was dissected to elucidate sub-themes. The research team meticulously scrutinized the proposed themes and sub-themes. Based on these discussions, themes and sub-themes were refined, merged, or separated to better represent the data and address the research questions. Table 2 outlines a consolidated view of the themes and their corresponding sub-themes that are pertinent to the research questions, as identified in the articles deemed suitable for inclusion. While quality appraisal is not typically a component of the scoping review methodology and highlighted by Arksey and O’Malley (2005), the analytical and review rigor in our study was upheld through a systematic, team-based approach of the PRISMA-ScR framework. This approach ensured thoroughness and accuracy, with the research team meticulously verifying each stage of the analysis as noted by Levac et al. (2010).

**Table 1.** Summary of the Selected Reviewed Studies.

Author(s)/year	Study aims	Country	Method	Study setting	Participants/sample size	Findings	Strengths and limitations
Aboderin (2007)	To better understand the perspectives and experiences of Nigerian trained nurses working in the U.K. nursing home and how these relate to conditions of globalization	United Kingdom	Exploratory qualitative	Nursing home	25 Nigerian trained RNs working in the independent nursing home sector	Loss in professional and social status upon migrating to the United Kingdom Struggles over authority and added to their feelings of professional marginalization Significant desire was expressed by most nurses to leave the nursing home sector and transition to hospital work in the NHS	Strengths: • Exploratory approach • Focus on globalization Limitations: • Participants self-reported
Allensworth-Davies et al. (2007)	To examine perceived organizational cultural competence in LTC facilities from the perspective of NAs, and to analyze its relation to job satisfaction across different countries of origin and racio-ethnic groups	United States	Qualitative	Four LTC facilities	135 NAs from four New England nursing homes	FB NAs might perceive their position as providing significantly more autonomy than do U.S.- born NAs Non-White NAs perceived their facilities as less culturally competent than did Whites, and also viewed their coworkers’ attitudes toward their race and culture more negatively Perception of organizational cultural competence was found to be the strongest predictor of job satisfaction among NAs Negative correlates where employees believing that different races and cultures were causing problems for the facility and that others didn’t want them to succeed because of their race or cultural background	Strengths • The study employs a robust empirical method • Focus on cultural competence Limitation • The cross-sectional nature of the study restricts its ability to establish causality between cultural competence and job satisfaction
Bourgeault et al. (2010)	To examine the role of immigrant care workers in the home and LTC sectors in Canada, focusing on their relationships with older adults and the implications for the quality of care	Canada	Qualitative design	Home and LTC sectors	77 immigrant care workers (RNs and practical nurses and personal support workers), 24 employers, and 29 older adults (current and future care recipients)	Some immigrant care workers felt comfortable developing very friendly relations with their older adult clients Some immigrant care workers are better care workers because their cultures foster a more positive attitude to caring for older adults Language sufficiency was a major issue affecting the quality of care	Strengths: • It provided valuable insights into the relations between immigrant care workers and older adults in Canada • In-depth interview Limitations: • The study was limited to three provinces • Possible generalizability issues
Khatutsky et al. (2010)	To examine and compare the characteristics, qualifications, training, language proficiency, working conditions, pay, benefits, and job satisfaction of immigrant and non-immigrant CNAs working in nursing facilities	United States	Quantitative: descriptive analysis	LTC settings (nursing homes)	The study linked 2004 data from the NNHS, the NNAS, the Area Resource File, Nursing Home Compare, and the Online Survey, Certification, and Reporting system. The NNHS sample included 1,500 nursing facilities, and a subsample of 790 was selected for the NNAS. A total of 4,542 CNAs were selected randomly to participate in the NNAS, yielding 3,017 completed interviews. The combined NNHS-NNAS response rate was 53%	About 51% of immigrant CNAs reported a primary language other than English, and a higher percentage had difficulty communicating with residents and staff due to language barriers Immigrant CNAs also consistently reported more positive attitudes and perceptions of their supervisors than non-immigrants Immigrant CNAs experience discrimination and language-related communication barriers at work	Strengths: • Large, nationally representative sample • Detailed analysis Limitation: • Outdated date (2004)
Sloane et al. (2010)	To provide a better understanding of the unique characteristics and challenges faced by immigrants’ nurses in the U.S. nursing home workforce	United States	Quantitative study: 2004 NNAS, conducted by the U.S. National Center for Health Statistics	U.S. nursing homes that participated in the NNHS	3,017 NAs	Immigrant felt significantly less respected by residents and families compared to U.S.-born NAs Immigrants were less likely to recommend nursing homework to others	Strengths: • Nationwide sample • Multivariate analysis Limitations: • The NNAS was not designed specifically to study immigrants
Walsh and O’Shea (2010)	To explore whether the marginalization of elder care in Ireland exacerbates the disadvantages experienced by migrant care workers To understand the effects, if any, on the working conditions of carers and the quality of care provided to older Irish individuals	Ireland	Mixed-method	Variety of settings across public, private, and voluntary institutional and home care facilities	40 migrant care workers (17 RNs and 23 care assistants) from a range of countries including India, the Philippines, the EU-accession countries (Poland, Latvia, Lithuania), Ukraine, and several African countries Surveys were also sent out to 570 long-stay care institutions and home care organizations, with a 50% response rate	Cultural misunderstandings, with 68% of employers seeing poor knowledge of Irish culture as a challenge Differences in approaches to care, such as the focus on clinical care over social care Language and communication barriers Experiences of discrimination among migrant carers, especially those from VM groups Many migrant care workers perceive their wages to be low, which negatively impacts staff morale and leads to high turnover	Strengths: • Data collection involved multiple sources • The study that gathers insights from various stakeholders offers a comprehensive view of the caregiving landscape Limitations: • Authors did not acknowledge any limitation for this study
Abrahamson et al. (2011)	To determine if race influences conflicts between nursing home staff, particularly CNAs, and family members of residents	United States	Quantitative	Multiple nursing homes selected from the 60 homes belonging to the New York Association of Homes and Services for the Aging	404 NAs who identified their race as either Black or White	Race did not significantly predict the level of conflict between NAs and family members Black NAs were not more likely to perceive poor treatment from residents’ families Black NAs were more likely to perceive dissimilar care expectations compared to the family members of the residents This perceived dissimilarity was found to influence perception of poor treatment and consequently resulted in increased staff/family conflict Increase in NA education was associated with increased staff/family conflict	Strengths: • Representative sample • The analysis provides a sophisticated means of assessing the relationship between variables Limitations: • Only focused on Black and White NAs • Race of the family members was not considered in the analysis
Adhikari and Melia (2015)	To examine the professional lives of Nepali migrant nurses working in the private nursing home in the United Kingdom	United Kingdom	Ethnographic	Private nursing home in the United Kingdom	21 Nepali nurses who had migrated to the United Kingdom	Nepali migrant nurses, who were highly qualified and had specialized experience, were typically employed in the LTC sector, providing personal care for elderly people, a phenomenon often referred to as “deskilling” Limited career choices hindered professional development opportunities, resulting in frustration, low job satisfaction, and decreased morale among the nurses International recruiters often misled Nepali nurses about job availability in the United Kingdom’s NHS, leading to mismatched expectations upon arrival Working primarily in elderly care and LTC settings led to a gradual loss of their specialized skills in management, teaching, and technical fields such as intensive care unit, critical care unit, midwifery, and other specialized areas	Strength: • Understudied demographic Limitation: • In-depth interview
Gao et al. (2015)	To understand the perceptions of the rewards and difficulties of residential aged care work among DCWs, and how these relate to their employment intentions	Australia	Qualitative	Non-profit RACF located in an urban area of Queensland, Australia	13 DCWs (seven NAs and six nurses) participated in the study	The demanding nature of care work was reported by all participants, with NAs focusing more on physical demands, and nurses on psychological demands The work–life balance facilitated by flexible work schedules and a multicultural working environment were viewed positively Organizational resources, such as supervisor support, co-worker interaction, opportunities for personal development, physical amenity, and staffing levels, were seen as crucial for staff retention	Strength: • Diverse sample Limitation: • Single RACF
Wagner, Brush, Castle, et al. (2015)	To investigate the communication challenges experienced by FB/FE nurses in U.S. nursing home settings	United States	Quantitative: cross-sectional, descriptive, comparative design	Nursing homes in five U.S. states	1,629 RNs and LPVNs working in 98 nursing homes across five states in United States completed the surveys	15.3% of respondents reported that others (including nurses, medical providers, residents, or family members) had difficulty understanding their use of the English language and/or accent Language and accent can act as a roadblock to effective communication	Strengths: • The inclusion of nurses from five U.S. states and various educational backgrounds enhances the diversity and representativeness of the sample • A multi-step approach was used to solicit participation, and the surveys were pilot tested to ensure reliability Limitations: • No linkages between culture, communication, and patient safety • The study didn’t account for the length of time FB nurses had spent in the United States, which could have influenced their language proficiency and accent • Study relies on self-reported data, which may be subjected to bias
Jenkins and Huntington (2016)	To examine the experiences of IENs (Filipino and Indian) who relocated to New Zealand to work as RNs in aged care	New Zealand	Qualitative descriptive	Single large retirement facility in urban New Zealand	Six IENs (one Indian and five Philippines)	Physical adjustment to distance from family and culture Feeling of cultural isolation Feeling of social isolation and loneliness Experience changes in practice settings; moving from acute care to nursing in aged care Socio-cultural differences between health systems Deskilling; unable to do more of nursing job Mastery in decision-making and people’s skills Unfamiliar with medical conditions associated with aged care setting Learn to understand New Zealand palliative care One of the greatest challenges of working in aged care is working with families of the residents	Strengths: • Participants review and modify their transcripts which enhances the credibility of the data • Purposive sampling method provided rich, detailed, and focused data Limitations: • Small participants • Recruitment from single aged care • Participants were primarily female and Filipino
Pung et al. (2017)	To investigate the elements of job satisfaction, evaluate the demands of immigration, examine the relationship between job satisfaction and immigration demands, and identify predictors of job satisfaction among international nursing staff working in LTC settings	Singapore	Quantitative: cross-sectional study	Two LTC institutions in Singapore	130 international nursing staff, including RNs, licensed practical nurses, and nursing aides who had worked for at least 1 year in the respective institutions and were non-Singaporeans	Around a third (32%) of the international nursing staff indicated that they were considering leaving their jobs Job satisfaction among the participants was at a moderate level, averaging 3.65 out of 5	Strengths: • Validated instruments • Appropriate statistical analyses Limitations: • Cross-sectional approach, not able to establish cause-and-effect relationships • Study relies on self-reported data, which may be subjected to bias
Livelo et al. (2018)	To investigate the knowledge, attitudes, and experiences of FARNs toward EOL care in the United States	United States	Qualitative descriptive	Hospital, LTC facility, rehabilitation center, hospice, and nursing home in the United States	15 FARNs	FARNs show compassion and patience, respect for patient’s wishes, and exhibit an understanding of the cultural dimensions of EOL care FARNs use self-awareness and cultural competency to effectively address EOL care challenges FARNs highlight the significance of family presence and kinship in EOL care, reflecting the cultural values of the Philippines FARNs confront conflicts and struggles while delivering EOL care, including decision-making about treatment cessation and managing emotional attachments with patients while maintaining professional boundaries Ability to facilitate communication helps them understand and respect patient wishes and family values	Strengths: • In-depth focus group discussions • Participants caring for older adults in various health care facilities Limitations: • Small sample size • Not sure if data saturation was reached
Willis et al. (2018)	To illustrate how the organizational ethics of two non-profit organizations aid in the integration of CALD staff into broader Australian society	Australia	Qualitative: interpretive design	Two non-profit RACFs	16 CALD staff (personal care assistant, ENs, RNs) from 12 countries	Harmonious work environment that encouraged good relationships and cultural diversity Various strategies to communicate with residents who couldn’t speak English Respect for elderly in the family as part of cultural belief They brought their own cultural values about elderly care and expressed positivity toward residential aged care in Australia	Strength: • Comprehensive data collection Limitations: • Limited participants • Challenge with cross-cultural communication
Schilgen et al. (2019)	To identify work-related stressors, resources, and coping strategies of migrant and minority nurses compared to autochthonous (native) nurses in the German homecare sector	Germany	Qualitative explorative	Nursing homes	24 migrant and 24 autochthonous nurses	Both migrant and autochthonous nurses perceived time pressure, patient lifting, lack of appreciation, and client’s personal fate as burdensome Language and cultural differences, along with prejudices and harassment faced by migrant and minority nurses, were notable barriers to collaboration within diverse nursing teams Migrant nurses found a sense of community in their shared migrant status as one of the coping strategies	Strength: • Strong methodology that provides valuable insights Limitation: • Selection bias due to the nursing managers providing the contact data of willing participants
Ham (2020)	To investigate the social processes influencing the workforce integration of first-generation immigrant health care professionals in a Dutch Nursing home	Netherlands	Ethnographic approach	Rural Dutch nursing home	20 participants (10 immigrants and 10 established nurses)	Some established health care professionals were skeptical about the professional abilities of the immigrant nurses Immigrant nurses felt that their Dutch colleagues did not consistently meet their expectations of professional care Language barriers were a significant issue for the immigrant nurses Racial bias in the professional setting	Strength: • Ethnography as a research method, which provides rich, in-depth data Limitation: • Small sample size may impact generalizability
Nursalam et al. (2020)	To capture and describe the lived experiences of Indonesian nurses working as care workers in Taiwan	Taiwan	Phenomenology	LTC facilities in Taiwan	16 Indonesian nurses working in care facilities in Taiwan	Taiwan’s restrictions on foreign nurses working as professional nurses led to a loss of professional identity and skills among Indonesian nurses as they ended up working in nursing homes and LTC facilities Language barriers posed a significant challenge to these nurses, impeding their ability to communicate effectively, voice their thoughts and opinions, and fulfill their roles effectively Limited career opportunities for these Indonesian nurses in Taiwan, contributing to their frustration and disappointment Supportive workplace environment Placement agencies also played a nurturing	Strength: • Understudied group Limitation: • Relatively small sample size
Oakley et al. (2020)	To explore the experiences of expatriate nurses providing EOL care to Muslim patients in the United Arab Emirates	United Arab Emirates	Qualitative descriptive	Palliative care unit of a 461-bed tertiary hospital in the United Arab Emirates	Nine expatriate RNs, each possessing a bachelor’s degree in nursing or equivalent, a valid home national license, basic life support, and 2 years’ clinical experience	Language was a significant barrier in providing EOLC Difficulty of mastering the Arabic language Relationships between nurses, patients, and families strengthened over time Nurses faced challenges managing support for families with limited time	Strength: • Provided insights into the lived experiences of expatriate nurses in a unique cultural context Limitation: • All participants were female
Rapp and Sicsic (2020)	To explore the contribution of immigrant workers to the LTC workforce	United States	Quantitative: data from the Annual Social and Economic Supplement of the Current Population Survey for years 2003 to 2019	LTC	LTC workers (nurses and personal care), both U.S.-born and FB 190,000 individuals interviewed nationwide	22.50% of the LTC workforce are FB Immigrant nurses help to maintain a stable supply of LTC services Immigrants have a 7.6-percent point increased probability of staying in the LTC workforce compared to U.S.-born citizens 10% increase in wages are correlated with an increase in the probability of staying in the LTC workforce for all categories of workers	Strengths: • Large sample size • Extensive timeframe of data collection Limitation: • Self-reported nature of the health status data
Solum et al. (2020)	To explore the experiences of FENs from EU/EEA countries with their preparation, orientation programs, and their first year of work in Norwegian elderly care institutions	Norway	Qualitative explorative	Four different elderly care institutions in Norway	Nine FENs from Poland, Lithuania, Latvia, Iceland, and Spain	FENs struggled to adjust to professional competence standards in their first year of work Deficiencies in preparation and orientation by recruitment agencies and institutions Language skills and communication challenges Cultural differences in nursing roles and social interactions at work	Strength: • Variety of participants providing diverse perspectives Limitation: • Small sample size
Syed (2020)	To examine how race and racialization emerge as social determinants of health in a LTC setting To explore the ways racism and racialization influence physical, mental, and social well-being	Canada	Mixed	LTC home in Ontario	Front-line care workers (nurses and personal support workers), managers, and ancillary staff	Over-representation of VM/racialized workers in front-line care roles Disparities in income were evident along racialized and class lines Interpersonal and structural forms of racism Discrimination from colleagues and residents	Strength: • Multiple data collection methods Limitation: • Single LTC home
Adebayo et al. (2021)	To gain an understanding of migrant care workers’ knowledge of dementia, care experiences, psychosocial well-being, and working conditions in RACFs	Australia	Quantitative: cross-sectional	RACFs	272 migrant care workers across five Australian states and one territory	Overall mean score of acculturation stress was 38.4 out of 75, with 38.9% of respondents scoring 40 or higher The nurses (ENs and RNs) had the highest acculturation stress levels when compared to other occupational roles in RACFs English proficiency was significant contributor to acculturation stress	Strength: • Investigates acculturation stress in migrant care workers Limitations: • Selection bias • Nonrandom and purposive sampling methods could affect the generalizability of the study findings
Angus et al. (2021)	To explore the challenges that IQNs in New Zealand face when providing palliative care in ARC facilities To comprehend how the specialist Palliative ARC nurse team can best support these IQNs in their roles	New Zealand	Qualitative descriptive studies (five focus group interviews)	ARC facilities	24 RNs among IQNs (20 women and 4 men) who are currently providing palliative care	IQNs experience challenges such as cultural differences, language barriers, and unfamiliarity with New Zealand’s palliative care practices and ARC systems IQNs also bring diverse skills and knowledge which enhance the care provided	Strengths: • Rigorous research method, qualitative thematic analysis • The use of FG for data collection Limitations: • Limited sample to nurses in a specific region • Unequal number of participants in each FG
Ham (2021)	To delve into the dynamics and intricacies of how first-generation immigrant nurses and native nurses collaborate in providing care to elderly residents in a Dutch nursing home	Netherlands	Ethnographic	Dutch nursing home	10 immigrant nurses (five men and five women) from East African countries, and 10 native nurses (10 women)	The immigrant nurses encountered language barriers, including difficulties with local language patterns, accents, and casual conversations, which made them feel self-conscious Limited exposure to the new cultural context They felt pressured to meet certain care standards Majority of residents expressed satisfaction with the care provided by immigrant nurses Unfamiliar context with health care Discrimination and prejudiced remarks Some residents refusing care from immigrant nurses Good mentoring relationships between immigrant and native nurses led to mutual learning and patient benefits	Strength: • The study offers insights into the challenges of workforce integration of immigrant nurses Limitation: • Its focus on the micro-level of personal interactions
Adebayo et al. (2023)	To explore migrant care workers’ perceptions of their job demands in RACFs, their coping strategies, and their intentions regarding staying or leaving the sector	Australia	Descriptive qualitative	RACFs	20 migrant care workers (4 RNs, 1 EN, 15 patient care assistant)	Cultural values, such as compassion and a history of caring for older family members, influenced participants’ decisions to work in RACFs Participants experienced various work-related stressors, including communication difficulties, discrimination, lack of familiarity with workplace routines, and challenges of working with residents with dementia Coping strategies employed by participants included ignoring stressors, seeking support from colleagues and family members, and engaging in meditation or spiritual practices Two-thirds of the participants expressed their intentions to continue working in RACFs Family-friendly workplaces, flexible working hours, and support from the organization were identified as factors that contribute to staff retention Perceived barriers to retention included tension and racially motivated treatment from non-migrant workers, disloyalty among migrant care workers, poor quality of care for residents, limited career options, and high workload	Strength: • Diverse sample of migrant care workers Limitation: • Study was conducted in one location

Note. NHS = National Health Service; LTC = long-term care; NAs = nursing assistants; CNAs = certified nursing assistants; NNHS = National Nursing Home Survey; NNAS = National Nursing Assistant Survey; DCWs = direct care workers; FB/FE= foreign-born/foreign-educated; IENs = Internationally Educated Nursing staff; FARNs = Filipino-American registered nurses; EOL = end-of-life; CALD = culturally and linguistically diverse; FENs = foreign-educated nurses; EU/EEA = European Union/European Economic Area; VM = visible minority; RACFs = residential aged care facilities; RNs = registered nurses; ENs = enrolled nurses; ARC = aged residential care; IQNs = internationally qualified nurses; LPVN = Licensed practical vocational nurse; FG= Focus Group.

**Table 2:** Outline of the Themes and Sub-Themes Derived From the Data.

Research Question 1: Experiences of IENs caring for older adults		
Theme	Sub-themes	Description (based on findings)
Cultural and social dynamics	Cultural understanding	Comfort with friendly relations; the positive cultural values of immigrant nurses regarding the care of older adults were linked to the development of amicable relationships with this demographic (Bourgeault et al., 2010; Livelo et al., 2018; Rapp & Sicsic, 2020). Cultural disparities are often associated with communication misunderstandings (Adhikari & Melia, 2015; Ham, 2021; Redfoot & Houser, 2008)
	Social isolation	Feelings of isolation and loneliness; challenges in cultural and social adjustments (Jenkins & Huntington, 2016)
Professional identity	Loss of status	Professional marginalization and desire to transition to other sectors (Aboderin, 2007; Adhikari & Melia, 2015; Ham, 2020; Nursalam et al., 2020)
	Deskilling	Employment in lower skilled roles leading to frustration and loss of specialized skills (Adhikari & Melia, 2015; Jenkins & Huntington, 2016; Nursalam et al., 2020)
Communication	Language and accent comprehension	Difficulties in communication negatively affecting care quality and workplace integration (Adebayo et al., 2021, 2023; Adhikari & Melia, 2015; Angus et al., 2021; Ham, 2020, 2021; Khatutsky et al., 2010; Nursalam et al., 2020; Oakley et al., 2020; Schilgen et al., 2019; Wagner et al., 2015; Wagner, Brush, Castle. et al., 2015)
Discrimination	Racial bias, prejudice, and inequality	Experiences of discrimination and prejudice in the workplace (Aboderin, 2007; Abrahamson et al., 2011; Adebayo et al., 2023; Ham, 2020, 2021; Khatutsky et al., 2010; Schilgen et al., 2019; Sloane et al., 2010; Syed, 2020; Walsh & O’Shea, 2010)
	Professional skepticism	Skepticism about professional abilities from colleagues (Ham, 2020)
Research Question 2: Factors facilitating or impeding IENs’ provision of high-quality health care services to older adults		
Facilitating themes	Sub-themes	Description (based on findings)
Organizational support	Cultural competence of facilities	Perceptions of organizational support and cultural competence were associated with job satisfaction (Allensworth-Davies et al., 2007; Angus et al., 2021; Gao et al., 2015; Willis et al., 2018)
	Mentorship and leadership	Supportive leadership and mentorship programs were associated with professional development (Adebayo et al., 2023; Ham, 2021)
Autonomy and conductive work environment		Autonomy in work perceived by foreign-born NAs associated with job satisfaction and better care (Adebayo et al., 2023; Allensworth-Davies et al., 2007; Gao et al., 2015; Wagner, Brush, Castle. et al., 2015; Willis et al., 2018)
Interpersonal skills	Relationship with patients and families	Strong relationships with patients and their families associated with improved care quality (Ham, 2021; Jenkins & Huntington, 2016; Khatutsky et al., 2010; Livelo et al., 2018; Oakley et al., 2020; Rapp & Sicsic, 2020; Walsh & O’Shea, 2010; Willis et al., 2018)
Impeding themes	Sub-themes	Description (based on findings)
Working conditions	Work-related stressors	High workload, communication difficulties, and work-related stress associated with poor job satisfaction and low retention (Adebayo et al., 2021, 2023; Allensworth-Davies et al., 2007; Gao et al., 2015; Jenkins & Huntington, 2016; Schilgen et al., 2019)
	Remuneration and benefits	More positive perceptions of wages were associated with high morale and lower turnover (Aboderin, 2007; Nursalam et al., 2020; Syed, 2020; Walsh & O’Shea, 2010)
Systemic issues	Credential recognition	Barriers due to credential recognition processes associated with poor job satisfaction (Adhikari & Melia, 2015)
	Immigration policies	Immigration demands and policies influencing job satisfaction and retention (Pung et al., 2017)

*Note.* IENs = Internationally Educated Nursing staff.

### Consultation

Arksey and O’Malley (2005) posited that while the consultation phase in Scoping studies is optional, its inclusion significantly enhances methodological rigor and should be viewed as an essential component (Levac et al., 2010). To fortify the robustness of our study, we actively sought the insights of key stakeholders well-versed in the care of older adults within LTC settings and distinguished scholars with a record of publications in this field. Our interactions included the dissemination of our preliminary findings and the incorporation of their seasoned expertise into our study’s framework.

## Results

### Study Characteristics

A total of 25 articles were thoroughly examined based on the preliminary review. They included 16 (64%) qualitative (Aboderin, 2007; Adebayo et al., 2023; Adhikari & Melia, 2015; Allensworth-Davies et al., 2007; Angus et al., 2021; Bourgeault et al., 2010; Gao et al., 2015; Ham, 2020, 2021; Jenkins & Huntington, 2016; Livelo et al., 2018; Nursalam et al., 2020; Oakley et al., 2020; Schilgen et al., 2019; Solum et al., 2020; Willis et al., 2018), 7 (28%) quantitative (Abrahamson et al., 2011; Adebayo et al., 2021; Khatutsky et al., 2010; Pung et al., 2017; Rapp & Sicsic, 2020; Sloane et al., 2010; Wagner, Brush, Castle. et al., 2015), and 2 (8%) mixed-method studies (Syed, 2020; Walsh & O’Shea, 2010). The studies were conducted in 12 different countries: the United States (*n* = 7; Abrahamson et al., 2011; Allensworth-Davies et al., 2007; Khatutsky et al., 2010; Livelo et al., 2018; Rapp & Sicsic, 2020; Sloane et al., 2010; Wagner, Brush, Castle et al., 2015), Australia (*n* = 4; Adebayo et al., 2021, 2023; Gao et al., 2015; Willis et al., 2018), the Netherlands (*n* = 2; Ham, 2020, 2021), New Zealand (*n* = 2; Angus et al., 2021; Jenkins & Huntington, 2016), the United Kingdom (*n* = 2; Aboderin, 2007; Adhikari & Melia, 2015), Canada (*n* = 2; Bourgeault et al., 2010; Syed, 2020), while Taiwan, Ireland, the United Arab Emirates, Germany, Singapore, and Norway (Nursalam et al., 2020; Oakley et al., 2020; Pung et al., 2017; Schilgen et al., 2019; Solum et al., 2020; Walsh & O’Shea, 2010) contributed one article each. In the study, IENs were predominantly employed in LTC settings such as nursing homes, home care facilities, residential aged care, retirement facilities, hospice, and palliative care unit. The majority of these nurses hailed from a diverse array of countries, including the Philippines, India, Nigeria, Poland, Nepal, Lithuania, Ukraine, Latvia, Indonesia, Iceland, and Spain. Within these LTC environments, IENs served in a variety of capacities, assuming roles ranging from RNs, LPNs/LVNs, NAs, or CNAs.

### Study Findings

In addressing Research Question 1, “Experiences of IENs in Caring for Older Adults,” the literature review revealed four major themes; (a) cultural and social dynamics; (b) professional identity; (c) communication; and (d) discrimination. Concerning Research Question 2, “Factors Facilitating or Impeding IENs’ Provision of High-Quality Health Care Services to Older Adults,” the finding encapsulated seven themes. The facilitating factors included (a) organizational support; (b) autonomy and a conducive environment; and (c) interpersonal skills. Conversely, the impeding factors were identified as (a) working conditions and (b) systemic issues (Table 2 outlines the themes and sub-themes derived from the data).

### Research Question 1, Experiences of IENs in Caring for Older Adults

#### Cultural and Social Dynamics

Cultural and social dynamics included two sub-themes: (a) cultural understanding and (b) social isolation.

##### Cultural Understanding

This sub-theme was exemplified by some immigrant care workers being comfortable developing friendly relations with older adult clients and reflecting positive cultural values toward caring for older adults (Bourgeault et al., 2010; Livelo et al., 2018; Rapp & Sicsic, 2020). Conversely, unfamiliarity with the cultural context of their new environment often led to misunderstandings (Adhikari & Melia, 2015; Ham, 2021). Livelo et al. (2018) detailed how foreign-educated and accredited registered nurses from the Philippines approached end-of-life care, highlighting the influence of cultural values on their care practices and the importance of cultural competency and knowledge adaptation. Redfoot and Houser (2008) identified cultural differences as a source of friction for IEN working in LTC settings. Due to their limited exposure to the new cultural context and informal language usage, it was occasionally difficult for immigrant nurses to engage in casual conversations with residents (Ham, 2021).

##### Social Isolation

Both the Adebayo et al. (2023) and the Jenkins and Huntington (2016) studies highlighted the sub-theme of social isolation as they reported challenges migrant health care workers faced when adjusting to a new country and culture. These challenges include physical adjustments to the new environment, feelings of social and cultural isolation, and adapting to different practice settings, particularly in aged care.

#### Professional Identity

The professional identity of migrant nurses is often marked by a loss of status and deskilling.

##### Loss of Status

Migrant nurses often experienced losses in professional and social status, including feeling underappreciated, unrecognized, and encountering limited career opportunities leading to professional marginalization and a desire to transition to other sectors (Aboderin, 2007; Adhikari & Melia, 2015; Ham, 2020; Nursalam et al., 2020).

##### Deskilling

A common issue is the phenomenon of “deskilling,” where highly qualified and experienced nurses were employed in lower skilled roles, leading to frustration (Adhikari & Melia, 2015; Jenkins & Huntington, 2016; Nursalam et al., 2020).

#### Communication

Across multiple studies, language barriers consistently appeared as a major issue for immigrant nurses, causing difficulties in communication with patients/residents, staff, and colleagues (Adebayo et al., 2021, 2023; Adhikari & Melia, 2015; Angus et al., 2021; Ham, 2020, 2021; Khatutsky et al., 2010; Nursalam et al., 2020; Oakley et al., 2020; Schilgen et al., 2019). In addition, Wagner, Brush, Castle et al., 2015 established that a language barrier existed between IENs, coworkers, residents, and their families. Communication difficulties due to language barriers impacted care quality and workplace integration (Adebayo et al., 2023; Angus et al., 2021; Bourgeault et al., 2010; Ham, 2020, 2021; Khatutsky et al., 2010; Nursalam et al., 2020; Oakley et al., 2020; Solum et al., 2020; Wagner, Brush, Castle et al., 2015; Walsh & O’Shea, 2010). Accent and cultural differences acted as roadblocks to effective communication (Schilgen et al., 2019; Wagner, Brush, Castle et al., 2015).

#### Discrimination

Two sub-themes were identified: (a) racial bias, prejudice, and inequity and (b) professional skepticism.

Several studies indicated that immigrant nurses experience discrimination and prejudice from both colleagues and patients or residents (Aboderin, 2007; Abrahamson et al., 2011; Adebayo et al., 2023; Ham, 2020, 2021; Khatutsky et al., 2010; Schilgen et al., 2019; Sloane et al., 2010; Syed, 2020; Walsh & O’Shea, 2010). This manifested in various ways, including disregard for their professional abilities, disrespect, and racial bias. Oakley et al. (2020) reported that relationships between nurses with their patients and patients’ families strengthened over time. Syed (2020) highlighted the over-representation of visible minority/racialized workers in challenging front-line care roles and suggested systemic inequities existed. The studies by Abrahamson et al. (2011) and Jenkins and Huntington (2016) highlighted the complexities of interacting with patients’ families in an aged care setting. IENs may face challenges due to dissimilar care expectations and the need to deal with the stress of working with families of the residents. Immigrant NAs felt less respected compared to U.S.-born NAs, although race did not significantly predict the level of conflict between NAs and family members (Abrahamson et al., 2011; Adebayo et al., 2023; Ham, 2020, 2021; Khatutsky et al., 2010; Sloane et al., 2010; Syed, 2020; Walsh & O’Shea, 2010). Another example of discrimination was that colleagues sometimes showed skepticism about the professional abilities of immigrant nurses (Ham, 2020).

### Research Question 2: Factors Facilitating or Impeding IENs’ Provision of High-Quality Health Care Services to Older Adults

In addressing Research Question 2, the literature revealed that while working with older adults, IENs encountered both positive and negative experiences. Facilitating themes revealed were (a) organizational support; (b) autonomy and conductive work environment; and (c) interpersonal skills.

#### Organizational Support

Positive experiences reported by immigrant nurses often revolve around having supportive work environments, nurturing relationships, good team collaboration, appreciative supervision, and management recognizing their competence (Aboderin, 2007; Ham, 2021; Nursalam et al., 2020; Schilgen et al., 2019) enhanced job satisfaction and care quality (Allensworth-Davies et al., 2007; Angus et al., 2021; Gao et al., 2015). This shows the importance of adequate support structures and diversity management in improving immigrant nurses’ experiences. Willis et al. (2018) showed how organizational values influence the experiences of culturally and linguistically diverse staff. The positive work environment facilitated by diversity management strategies such as buddy systems, assistance programs, and flexible arrangements enabled staff to adapt to their roles and develop unique approaches to care of older adults. Rapp and Sicsic (2020) demonstrated a higher retention rate and better self-reported health among immigrants compared to U.S.-born citizens in the LTC workforce, indicating the significant contributions of immigrants in this sector. Gao et al. (2015) and Allensworth-Davies et al. (2007) also highlight the importance of organizational resources, such as supervisor support and opportunities for personal development, in influencing job satisfaction and retention among health care workers, whereas some IENs indicated they were considering leaving their jobs (Pung et al., 2017). Adebayo et al. (2023) uncovered motivations for working in residential aged care facilities, such as cultural values and training opportunities. Supportive leadership and mentorship programs were key in enhancing professional development (Ham, 2021).

#### Autonomy and Conductive Work Environment

Workplace autonomy and flexible work schedules contributed to job satisfaction and better care. A multicultural working environment was viewed positively (Adebayo et al., 2023; Allensworth-Davies et al., 2007; Gao et al., 2015; Willis et al., 2018). IENs’ diverse skills enhanced care provided, contributed to mutual learning environments, and often led to more positive perceptions of their supervisors and workplaces (Wagner et al., 2020).

#### Interpersonal Skills

Building strong relationships with patients and families improved care quality. Immigrant CNAs reported more positive attitudes and perceptions of their supervisors than non-immigrants, with most residents expressing satisfaction with the care provided by immigrant nurses (Ham, 2021; Khatutsky et al., 2010; Livelo et al., 2018; Oakley et al., 2020; Willis et al., 2018). IENs improved their decision-making and interpersonal skills, as well as their preconceived notions and beliefs about elderly care (Jenkins & Huntington, 2016), and also developed the ability to respect patients’ wishes while acting in their best interests (Livelo et al., 2018). Walsh and O’Shea (2010) provided valuable insights into the significant presence of migrant workers in Ireland’s elder care sector. Migrant workers brought with them professional training, high skills, reliability, and low absenteeism, maintaining a stable supply of LTC services (Rapp and Sicsic, 2020) contributing to the quality of care.

Conversely, impeding themes related to difficult working conditions and systemic issues.

#### Working Conditions

High workload, communication difficulties, and stress led to reduced job satisfaction and retention, with physical and psychological demands exacerbating these challenges (Adebayo et al., 2021, 2023; Allensworth-Davies et al., 2007; Gao et al., 2015; Jenkins & Huntington, 2016; Schilgen et al., 2019). Most IENs encountered challenges including cultural differences, difficulties adapting to the new style of living, and increased time needed to understand new technologies at places of work. These struggles could lead to misunderstandings and feelings of under-preparedness, indicating the need for better preparation, mentorship, and support systems for foreign-educated nurses. The acculturation process can be stressful (Adebayo et al., 2021), particularly for nursing professionals who have to adjust to socio-cultural differences in health systems and unfamiliar medical conditions. Walsh and O’Shea (2010) discuss the cultural misunderstandings that can arise due to these differences and the need for more support and cultural induction programs for migrant care workers.

Inadequate compensation impacted morale and intent to stay, leading to high turnover. Income disparities were evident along racialized and class lines (Syed, 2020; Walsh & O’Shea, 2010). Many of the nurses migrated with the hope for better pay, improved living standards, and career development opportunities (Aboderin, 2007; Nursalam et al., 2020). However, these expectations often clash with the reality they face in their host countries, leading to feelings of disappointment, frustration, and a desire to change their employment conditions.

#### Systemic Issues

Delays in credential recognition and mismatched expectations due to misinformation by international recruiters affected job roles and satisfaction (Adhikari & Melia, 2015). Mismatched expectations among IENs are often due to misinformation provided by international recruiters, coupled with limited career opportunities. Stringent immigration policies contribute to challenges in achieving job stability and satisfaction for IENs (Pung et al., 2017).

## Discussion

This article presents a comprehensive review of relevant literature on IENs working with older adults in LTC settings. Following our in-depth engagement with the literature, we conclude this is the first Scoping study explicitly focused on IENs caring for older adults. The purpose of this article was to conduct a Scoping study of the literature to determine what evidence exists regarding IENs’ experiences caring for older adults and to synthesize study findings. The United States continues to grapple with a nurse shortage and an aging nursing workforce (Budden et al., 2013). IENs have stepped in to play an essential role in the U.S. nursing workforce, aiding in creating a more diverse population of RNs that better reflects the evolving diversity of U.S. patients. Insights gained from a Scoping study shed light on the experiences of IENs, particularly in high-income nations and settings focused on the care of older adults. The review paints a comprehensive picture of the challenges that IENs face, their experiences, and factors impacting their service delivery. It’s evident from the findings that IENs encounter numerous difficulties as they transition from their home countries to live in high-income nations and care for older adults. The findings suggest that IENs face hurdles, including cultural differences, language barriers, social isolation, discrimination, professional marginalization, and unfamiliarity with LTC facilities and geriatric care. The study indicates that additional support and training could help mitigate these challenges and harness the advantages they offer. Wolcott et al. (2013) proposed a variety of orientation programs to assist in this transition, focusing on elements like language acquisition, communication, understanding the structure, philosophy, and operation of the U.S. health care system. Also, they suggested learning about policies and procedures specific to different settings, cultural orientation to the United States and culturally appropriate behaviors, detailed guidance on new tools and technologies, and refresher courses at 6 months and 1 year after the initial orientation (Wolcott et al., 2013).

The ability to deliver high-quality nursing care services is a key attribute of professional nurses. Organizational support, autonomy, and conductive work environment and strong interpersonal relationships with patients and families also emerge as a factor that enhances care quality (Ham, 2021; Khatutsky et al., 2010; Livelo et al., 2018; Oakley et al., 2020; Willis et al., 2018). From the insights drawn from this Scoping study, it is apparent that numerous elements influence an IEN’s ability to provide quality health care services. Obstacles that hinder IENs from realizing their full potential in delivering quality care to older adults encompass challenges with language and accent comprehension, cultural attitudes and beliefs, as well as experiences of discrimination and prejudice. Connor and Miller (2014) contend that subpar communication skills can erode self-confidence and foster increased work-related stress. Liou and Cheng (2011) affirm that deficiencies in language and communication skills pose a barrier to nurses in providing high-quality nursing care services. To elevate care standards, K. Iheduru-Anderson (2020), K. C. Iheduru-Anderson et al. (2021), and Xue (2015) argue that IENs need to acquire communication skills that are attuned to the host culture and new work environments, in addition to fostering proper integration. Allen (2018) suggests that nursing leaders can motivate IENs to engage in community outreach endeavors, which would enable them to familiarize themselves with various cultures and understand them beyond their workplace setting. Such activities can aid in overcoming accent and communication barriers, thus contributing to improved patient care. Baptiste (2015) explored the impact of workplace discrimination on IENs, revealing that such discrimination can compromise patient safety, satisfaction, and the provision of high-quality health care services. Discrimination at the workplace, particularly of a racial nature, can adversely affect the lifestyle of IENs and hinder the delivery of superior health care services (K. C. Iheduru-Anderson et al., 2021; Xue, 2015). Workplace discrimination introduces an extra layer of stress for IENs and is seen as an obstacle to career advancement and professional acknowledgment (Baptiste, 2015). Communication obstacles, discrimination, and feelings of alienation further exacerbate work-related stress (Connor & Miller, 2014). IENs caring for older adults navigate a complex terrain marked by both enriching encounters and formidable barriers. The studies underscore the need for robust support systems, cultural induction initiatives, and structural reforms to optimize the integration and well-being of IENs in the LTC settings.

### Limitations

Although this review contains numerous findings that contribute to the body of knowledge regarding IENs caring for older adults, the findings should be interpreted cautiously for the following reasons. First, the articles examined for this review hail from a total of 12 different nations, with 7 out of the 25 reviewed originating from the United States. The remaining articles were sourced from a variety of countries, each representing diverse cultures. This variability may impair the findings’ generalizability. Second, several studies included RNs, LPNs/LVNs, CNAs, and NAs in their samples, resulting in a lack of professional specificity. Variations in educational backgrounds and professional responsibilities among nursing staff may exist; hence, caution is advised against generalizing the study findings. Although this Scoping study was limited to peer-reviewed articles published up until 2023, it entailed a thorough and detailed search process. Despite these efforts, there is a possibility that certain pieces of literature could have been missed.

### Direction for the Future Research

Current gaps in knowledge have several implications for clinical intervention, policymakers, and researchers. Future research should aim to investigate these themes in more depth, particularly in understanding how to better support IENs working in LTC in their professional development and integration into the new cultural environment, how to improve their working conditions, and strategies for mitigating language and cultural barriers. Understanding and addressing these issues are crucial for improving the quality of care provided to older adults, ensuring job satisfaction among IENs and maintaining a sustainable health care workforce. This review reveals that among the seven U.S. articles reviewed, only one is a qualitative study. The sole qualitative study from the United States suggests a need for additional research on personal narratives to delve into the real-life experiences of IENs providing care for the older adults. It is noteworthy that many articles in this review involve samples from diverse nursing staff, leading to a lack of professional specificity. Hence, there is a need for research that specifically explores the unique experiences of RNs working in LTC settings.

## Conclusion

This review has provided a thorough examination of the literature pertaining to IENs in LTC settings, presenting insights into their unique challenges and contributions. Despite the hurdles IENs face, such as language barriers, cultural differences, and potential discrimination, their diverse skills and experiences offer a valuable enhancement to the quality of care for older adults in high-income countries. The narrative analysis indicates a pressing need for comprehensive support and training programs, to better integrate IENs into their new professional and cultural environments. Employers should provide supportive work environments and ensure that professional skills and competencies of IENs are recognized and utilized to the full extent. Furthermore, understanding and appreciating the unique skills and experiences that IENs bring to their work can be beneficial for both the workforce and the older adults they serve. Moreover, the paucity of qualitative studies within the U.S. context emphasizes the need for further research into IENs’ lived experiences. The insights gathered can inform interventions by clinical practitioners, policymakers, and researchers to improve care quality and boost job satisfaction among IENs in LTC. Emphasizing inclusivity and recognition of IENs’ professional skills is crucial, highlighting a collective responsibility among health care sectors, policymakers, and society at large to foster a supportive environment for IENs.
